# Examining the Directional Effects of Insomnia, Anxiety, and Depression Symptoms: Results From a Longitudinal Study Conducted During the COVID‐19 Pandemic

**DOI:** 10.1002/jclp.70207

**Published:** 2026-08-11

**Authors:** Jamie Walker, Ana J. Bridges, Alexandria Muench, Michael L. Perlis, Ivan Vargas

**Affiliations:** ^1^ Department of Psychological Science University of Arkansas Fayetteville Arkansas USA; ^2^ Behavioral Sleep Medicine Program University of Pennsylvania Philadelphia Pennsylvania USA; ^3^ Department of Psychology University of Notre Dame Notre Dame Indiana USA

**Keywords:** anxiety, COVID‐19, depression, insomnia

## Abstract

**Objectives:**

While some research suggests that increases in anxiety symptoms drive changes in insomnia and depression symptoms, other studies support that these relations may be bidirectional. The present study therefore aimed to further examine how anxiety, insomnia and depression symptoms predict changes in these symptoms over time.

**Methods:**

Participants were recruited via an online participant database, social media advertisements, and other online sites, resulting in a large sample (*N* = 2,666) of adults from across the United States. Well validated instruments were used to measure symptoms of depression (CES‐D), anxiety (STAI), and insomnia (ISI). Data were collected via a series of Qualtrics surveys administered online at two time points: March–June 2020 (baseline) and January–March 2021 (follow‐up).

**Results:**

Insomnia symptoms (*β* = 0.142, *p* < 0.001) but not anxiety symptoms (*β* = 0.019, *p* = 0.445) significantly predicted depression symptoms. Depression symptoms (*β* = 0.077, *p* = 0.001) but not anxiety symptoms (*β* = 0.039, *p* = 0.075) significantly predicted insomnia symptoms. Both insomnia (*β* = 0.060, *p* = 0.001) and depression symptoms (*β* = 0.281, *p* < 0.001) significantly predicted anxiety symptoms. Although multiple cross‐lagged paths were statistically significant, effect sizes were generally small, and no single pathway was meaningfully stronger than others. Additionally, secondary exploratory mediation models revealed significant indirect effects between anxiety and depression through insomnia (model 1 indirect *β* = 0.027, *p* = 0.007; model 2 indirect *β* = 0.008, *p* = 0.036).

**Conclusions:**

Findings suggest that insomnia symptoms were prospectively associated with subsequent depressive and anxiety symptoms. Depression symptoms also predicted later anxiety symptoms, highlighting the complex longitudinal associations among these symptom domains. However, results did not support a temporal pathway in which anxiety precedes the development of comorbid insomnia. Future studies should assess temporal order more closely, using high density longitudinal sampling strategies (i.e., more than two data collection points) to further clarify the temporal ordering of these symptom domains.

## 1 Introduction

Depression is a highly prevalent disorder with large public health and economic impacts (Bloom 2004; Marcus et al. 2012). Approximately 10.4% of adults in the United States experienced at least one episode of major depressive disorder (MDD) per year (Hasin et al. 2018). Mean healthcare utilization costs have been found to be 3.5 times higher among depressed patients than non‐depressed patients, and mean social services costs (e.g., rent assistance, employment/income assistance) three times higher (Tanner et al. 2020). MDD is also related to or considered a risk factor for several other psychological and physical health issues, including suicidal ideation and attempts (Ribeiro et al. 2018), cardiovascular disease (Rajan et al. 2020), and cancer mortality (Wang et al. 2020). Additionally, clinical depression is the most common psychiatric disorder in individuals who die by suicide (Henriksson et al. 1993). It is therefore critical that research efforts focus on identifying the factors that contribute to the development of MDD (i.e., risk factors). This is even more important because MDD is often difficult to treat, due to issues related to complex comorbidities (Steffen et al. 2020), low treatment compliance (Gaynor et al. 2006), and inflexible maladaptive thought and behavior patterns (McCullough 2003). For example, approximately one third of depressed patients experience treatment‐resistant depression, accounting for a disproportionately large burden (e.g. increased disability, financial costs, human suffering, suicide) (Ionescu et al. 2015). Identifying risk factors for depression may highlight potential targets for prevention or early intervention efforts that may reduce the overall burden of the disorder.

Many studies have examined temporal relationships among symptoms of depression, anxiety, and insomnia, although much of this work has focused on pairs of constructs rather than modeling all three simultaneously within a single longitudinal framework. For example, prior research consistently supports that the onset of MDD is predicted and preceded by pre‐morbid symptoms of generalized anxiety (Cole et al. 1998; Jacobson and Newman 2016; Rice et al. 2004; Blake et al. 2018; Johnson et al. 2006). Furthermore, studies have shown that elevated anxiety symptoms predict future onset of a depressive episode (Fava et al. 2000; Keenan et al. 2009; Starr and Davila 2012). While symptoms of anxiety and depression are often comorbid, several longitudinal studies have found that anxiety symptoms often precede the onset of MDD, even after controlling for prior depression symptoms (Cole et al. 1998; Rice et al. 2004). For example, Cole and colleagues’ findings were based on results from depression and anxiety questionnaires administered every 6 months for 3 years. Another study demonstrated that heightened anxiety symptoms in adolescence predicted clinical depression (assessed at 12–14 years later) in adulthood (Jacobson and Newman 2016). Additionally, results from a meta‐analysis looking at the longitudinal relation between anxiety and depression supported that, while the link between anxiety and depression symptoms is bidirectional, there is generally more support that anxiety symptoms predict future depression symptoms (Jacobson and Newman 2017). Furthermore, the temporal order of anxiety and depression symptoms may vary by anxiety type (general vs panic vs social), however, there is a lack of prior studies differentiating the different types of anxiety (Long et al. 2018). Therefore, a better understanding of the mechanisms by which anxiety symptoms lead to depressive symptoms (and subsequently MDD) is an important area of research. For example, anxiety is associated with feelings of inhibition, nervousness, and reluctance to interact with others (Leary and Kowalski 1995; Russell and Topham 2012), which likely interferes with one's ability to connect with potential sources of support and could lead to feeling disconnected, lonely, and hopeless—symptoms known to be related to depression.

Similarly, prior research consistently supports that MDD is associated with both pre‐morbid insomnia symptoms, as well as insomnia disorder. In one cross‐sectional study, those with insomnia disorder were nearly ten times more likely to have clinically significant depression than those without insomnia disorder (Taylor et al. 2005). Additional cross‐sectional analyses have indicated that individuals with insomnia disorder report more severe or persistent symptoms of depression than individuals without insomnia disorder (Mason and Harvey 2014; Pigeon et al. 2008; Taylor et al. 2005). Longitudinal studies have also found that symptoms of insomnia precede the onset of MDD when assessed after a variety of follow‐up periods ranging from just a few months to over several decades (Baglioni et al. 2011; Hertenstein et al. 2019; Li et al. 2016), including comorbid cases of insomnia disorder and MDD (Alvaro et al. 2013; Baglioni et al. 2011; Mason and Harvey 2014). Similarly, a cross‐lagged path analysis showed that insomnia symptoms predicted subsequent depression symptoms at follow‐up 1 year later (Raman et al. 2022). Additionally, a meta‐analysis of longitudinal studies showed that non‐depressed individuals with insomnia disorder have a two‐fold risk of developing depression, compared to people with no sleep difficulties (Baglioni et al. 2011). Taken together, these data provide support that insomnia disorder is a separate condition that precedes and is a risk factor for MDD and not simply a symptom of the disorder.

The COVID‐19 pandemic has had a major impact on sleep and psychological well‐being, with symptoms of anxiety, depression, and insomnia increasing in their prevalence throughout the pandemic (Alzueta et al. 2023; Chu et al. 2023; Machado et al. 2023; Mahmud et al. 2023; Morin et al. 2021; Wang et al. 2021). Social distancing, and the resulting self‐isolation, has had negative consequences on sleep and mental health (Halsøy et al. 2021; Mandelkorn et al. 2021; Pilcher et al. 2022; Vargas et al. 2021). According to one study, for example, healthcare workers experienced increased negative mood and sleep disturbances during the outbreak, stressing the need to establish ways to mitigate mental health risks and adjust interventions under highly stressful conditions—like a global pandemic (Pappa et al. 2020). Another study found that individuals who were quarantined during the pandemic had higher levels of depression, anxiety, and insomnia symptoms than individuals who did not quarantine (Wang et al. 2021). The COVID‐19 pandemic was associated with widespread increases in anxiety, insomnia, and depressive symptoms, creating a context in which these clinically relevant constructs could be examined prospectively. Rather than serving as the primary source of novelty, the pandemic context provides an opportunity to evaluate how these symptoms relate to one another during a period characterized by elevated stress and uncertainty.

Although there is robust evidence supporting that there is a strong association between symptoms of anxiety, insomnia, and depression (e.g., high comorbidity rates), the temporal relationship among all three is less clear. One of the few studies to date that has examined the temporal relationship among all three studied a sample of adolescents (Johnson et al. 2006). Here they found that among those with comorbid disorders, anxiety disorders preceded insomnia disorder 73% of the time, and insomnia disorder preceded MDD 69% of the time. Any prior anxiety disorder was associated with an increased risk of insomnia disorder, but prior insomnia disorder was not significantly associated with onset of an anxiety disorder. In addition, another study of adolescents found that insomnia symptoms mediated the longitudinal relationship between symptoms of anxiety and depression (Li et al. 2018). Similarly, a longitudinal study of individuals from Sweden found that elevated insomnia symptoms were associated with an increased risk for developing depression (but not anxiety) symptoms 6 months later. They also found that insomnia symptoms predicted persistent depression symptoms at the follow‐up (i.e. those with symptoms of insomnia and depression at baseline were more likely to continue to have symptoms of depression 6 months later) (Johansson et al. 2021). In addition, one systematic review suggested that insomnia symptoms are bidirectionally related to symptoms of anxiety and depression (Alvaro et al. 2013).

Based on prior longitudinal findings, one plausible possibility is that insomnia symptoms may be involved in the association between anxiety and later depressive symptoms. However, multiple pathways among these constructs are possible, including bidirectional associations and alternative temporal sequences. The present study therefore examined prospective relationships among anxiety, insomnia, and depression without assuming that any single pathway would necessarily be supported.

Although insomnia symptoms have historically been considered a component of depression, contemporary research and diagnostic frameworks increasingly distinguish insomnia as a separate clinical construct with unique etiological pathways. Longitudinal studies demonstrate that insomnia frequently precedes the onset of depression and confers risk above and beyond shared variance with mood symptoms. Insomnia also shows distinct patterns of persistence, treatment response, and biological mechanisms, suggesting it functions as an independent disorder rather than merely a manifestation of depressive symptomatology. Clarifying this distinction is essential for framing insomnia as a potential causal or maintaining factor in the broader anxiety–depression symptom network.

Across prior longitudinal studies examining temporal associations among anxiety, insomnia, and depression, assessment timing has varied considerably. Many investigations have used widely spaced follow‐up intervals, often between 6 and 12 months or longer (e.g., Johansson et al. 2021; Johnson et al. 2006; Li et al. 2018). While these designs have contributed important evidence, the limited temporal resolution of low‐frequency assessments constrains the ability to draw precise conclusions about temporal ordering. The present study's timing (approximately 9–12 months between assessments) is consistent with this existing literature but is subject to the same constraints inherent in two‐wave research designs.

The current study's primary aim was to examine the temporal, cross‐lagged associations among anxiety, insomnia, and depressive symptoms during the COVID‐19 pandemic. As a secondary, exploratory analysis, we evaluated whether insomnia symptoms statistically mediated the prospective association between anxiety and depressive symptoms. This mediation analysis was included to test one plausible indirect effect suggested by prior longitudinal research. While these associations are likely bidirectional, there is evidence that both anxiety and insomnia predict future depression. Furthermore, theoretical models of insomnia suggest that anxiety and hyperarousal at night perpetuates insomnia over time (i.e., leads to the transition from acute to chronic insomnia; Perlis et al. 2010). Therefore, it is plausible that insomnia may represent one pathway through which anxiety symptoms are associated with later depressive symptoms, particularly in the context of prolonged stress.

The association between anxiety, insomnia, and depression symptoms has been well documented, yet relatively few studies have examined their prospective relations simultaneously within a single longitudinal framework. Existing adult longitudinal studies have often focused on pairs of constructs (e.g., anxiety and depression or insomnia and depression) or have used different analytic approaches, leaving important questions regarding their relationships unresolved.

The current study examined this in a sample of adults in the U.S. during different points in the first year of the COVID‐19 pandemic. Analyses were performed on longitudinal data to assess the temporal relations among anxiety symptoms, depression symptoms, and insomnia symptoms during the COVID‐19 pandemic. Our hypotheses include: (1) greater baseline anxiety symptoms will be associated with greater depression symptoms at follow‐up while controlling for baseline depression and insomnia symptoms, (2) greater baseline anxiety symptoms will be associated with greater insomnia symptoms at follow‐up while controlling for baseline depression and insomnia symptoms, and (3) greater baseline insomnia symptoms will be associated with greater depression symptoms at follow‐up while controlling for baseline depression symptoms. This study highlights the importance of addressing sleep and mental health concerns, which has been critical during a global pandemic, however, these data have implications for the link between symptoms of anxiety, insomnia, and depression beyond the COVID‐19 pandemic. Specifically, clarifying the prospective associations among these symptoms may inform prevention and intervention efforts. If certain symptoms consistently predict increases in other forms of distress over time, they may represent useful targets for early intervention.

## 2 Method

All procedures were performed in compliance with relevant laws and institutional guidelines and have been approved by the appropriate institutional committees at the University of Arkansas and the University of Pennsylvania. Informed consent was obtained, and the privacy rights of human subjects were observed.

### 2.1 Study Design and Scope

The current study is a longitudinal, observational investigation designed to examine temporal relationships among anxiety, insomnia, and depressive symptoms. While these findings may inform future prevention or intervention strategies, the study does not include any treatment manipulation or evaluation of therapeutic efficacy. Consequently, all analyses are intended to test etiological and predictive relationships rather than treatment effects.

### 2.2 Participants and Procedure

The original sample included 4,145 adult participants (*M*_age_ = 45.8, SD_age_ = 16.6; 78.7% female; 82.7% white) recruited from the United States to participate in an online study on social distancing, sleep, and mood. Study recruitment began during the first 3 months following the start of the COVID‐19 pandemic (COVID‐19 was declared a pandemic in March 2020; CDC 2022). Convenience sampling strategies were used, such that recruitment occurred through postings on multiple social media websites (e.g., Facebook, Reddit), online newsletters, and ResearchMatch. Participants were included in the study if they were at least 18 years old, had access to the Internet, and were able to read and write in English. The sample included participants from across the United States, including all geographic regions (% by region: 27.5 Southeast, 26.7 Midwest, 23.5 Northeast, 12.6 West, 5.6 Southwest, 4.1 Rocky Mountains). The sample also included the following distribution of highest level of education: 0.3% less than high school, 4.5% high school graduate, 13.6% some college, 7.2% 2‐year degree, 36.1% 4‐year degree, 30.5% professional degree, and 7.8% doctoral degree.

Eligible individuals were first invited to complete a baseline survey (Time 1 or T1). This brief (less than 30 min) online baseline survey was administered March–June 2020 via Qualtrics XM (Provo, UT) and assessed social distancing, mood, sleep, physical activity, and basic demographic information. A follow‐up survey (Time 2 or T2) was administered 10–12 months following the initial survey (i.e., January–March 2021). The follow‐up survey consisted of similar items but also included more specific questions regarding COVID‐19 testing and symptoms. Of those participants that were given the initial baseline survey, 94.3% (*n* = 3909) completed all study measures and were used in the present analyses. Of those participants with complete baseline data, 31.8% (*n* = 1243) were lost to follow‐up (i.e., did not have complete symptom data). Therefore, the final sample (i.e., those that completed surveys at both baseline and follow‐up) included 2666 participants between the ages of 18 and 86 years old (*M*_age_ = 47.0, SD_age_ = 16.7; 79.0% female; 84.1% white). The final sample, compared to those participants that were lost to follow‐up (LTFU), was older (*M*_age_ in years, final sample = 47.0, LTFU = 42.8 years), had a greater percentage of participants who identified as white (%, final sample = 84.1, LTFU = 79.2), were married (%, final sample = 46.1, LTFU = 39.1), and were college‐educated (%, final sample = 82.7, LTFU = 75.0). The final sample did not vary regarding gender or employment status (i.e., unemployed, employed part‐time, employed full‐time).

### 2.3 Measures

#### 2.3.1 Depressive Symptoms

The CES‐D is a 20‐item measure that asks individuals to rate how often over the past week they experienced symptoms associated with depression, such as restless sleep, poor appetite, and feeling lonely Weissman et al. 1977). Response options range from 0 to 3 for each item (0 = *Rarely or None of the Time*, 1 = *Some or Little of the Time*, 2 = *Moderately or Much of the time*, 3 = *Most or Almost All the Time*). Scores range from 0 to 60, with high scores indicating greater depressive symptoms. CES‐D scores ≥ 16 were used to indicate clinically elevated depressive symptoms, consistent with the original validation and widespread use of the scale in epidemiological research (Radloff 1977). This measure is widely used and has demonstrated excellent validity, which speaks to previous research on the psychometric properties of this scale. The CES‐D was administered at baseline and follow‐up and demonstrated internal consistency (Cronbach's alpha = 0.93) for this sample at baseline.

#### 2.3.2 Anxiety Symptoms

The state anxiety subscale of the State‐Trait Anxiety Inventory (STAI) is a validated 20‐item self‐report questionnaire, with responses given on a 4‐point scale to assess state anxiety (Spielberger et al. 1983). Various reliability and validity tests have been conducted on the STAI and have provided sufficient evidence that the STAI is an appropriate and adequate measure for studying anxiety in research and clinical settings (Sesti 2000). State anxiety (as opposed to trait anxiety which can also be measured by the STAI) was chosen as a variable due to its classification as a potential contributor to the development and maintenance of insomnia symptoms (Taylor et al. 2005). STAI‐State scores ≥ 40 were used to indicate clinically elevated anxiety symptoms, consistent with prior research and normative guidelines (Spielberger et al. 1983). The STAI was administered at baseline and follow‐up and demonstrated internal consistency (Cronbach's alpha = 0.93) for this sample at baseline.

#### 2.3.3 Insomnia Symptoms

The Insomnia Severity Index (ISI) is a 7‐item self‐report measure designed to assess perceived severity of insomnia (Morin 1993). Each item uses a Likert scale from 0 (*not at all satisfied*) to 4 (*very much satisfied*). The items are summed, yielding a minimum score of 0 and a maxi‐mum score of 28. Consistent with established cut points, ISI scores ≥ 8 indicate at least subthreshold insomnia symptoms, whereas scores ≥ 15 reflect clinically significant insomnia (Morin et al. 2011). The ISI was administered at baseline and follow‐up and demonstrated internal consistency (Cronbach's alpha = 0.89) for this sample at baseline.

### 2.4 Consideration of Sleep Item in CES‐D and Distinctiveness of Insomnia

The CES‐D includes one item assessing restless sleep, which may partially overlap with the construct measured by the ISI. This overlap could slightly inflate correlations between depressive and insomnia symptom scores. However, prior research indicates that the ISI captures insomnia severity as a construct that is largely distinct from depression and anxiety, both psychometrically and conceptually (e.g., Bastien et al. 2001; Morin et al. 2011). Sensitivity analyses in prior longitudinal studies show that the predictive relationship between insomnia and depression persists even when overlapping sleep items are accounted for. Therefore, while some overlap exists, the ISI provides a reliable measure of insomnia symptoms independent of mood symptoms, supporting its use in testing the proposed temporal and mediational relationships in this study.

### 2.5 Statistical Analyses

Statistical analyses were performed using R version 4.2.0. An auto‐regressive cross‐lagged panel model was estimated using T1 and T2 scores for anxiety, insomnia, and depression. This involved regressing the T2 score of each variable on its own T1 value and that of the other two variables. T1 scores for each variable were then covaried with T1 scores of the other two variables. Additionally, the error terms for the T2 scores of each variable were covaried with that of the other two variables.

Because all constructs were represented using total scores from validated self‐report measures rather than latent variables, no separate measurement model was estimated. This approach is consistent with the majority of cross‐lagged research using symptom scales; however, it also means that measurement error could not be explicitly modeled. As a result, the estimates reflect observed score associations and assume that the total scores adequately capture the intended constructs without additional factor analysis. The cross‐lagged model was estimated on the variance‐covariance matrix and used full information maximum likelihood FIML by default, thereby avoiding the problem of listwise deletion of incomplete cases in the raw data set.

An auto‐regressive cross‐lagged panel model was chosen over a mediation model due to only having two time points of data collection, as opposed to the three time points required for a true mediation model. Although research questions regarding indirect effects often use mediation analyses with only one or two time points, an auto‐regressive cross‐lagged approach provides the best representation for how these data were collected and examines the downstream (as opposed to co‐occurring) effect of these variables on one another. In other words, it allows examination of prospective associations among symptoms of anxiety, insomnia, and depression, while accounting for prior symptom levels.

Indirect effects of anxiety on depression through insomnia were also calculated as a secondary exploratory analysis. Because a true longitudinal mediation model requires at least three assessment points, the present analyses were not intended to test mediation, causal mechanisms, or temporal sequencing among the constructs. Instead, the indirect effects represent descriptive combinations of estimated regression paths within the cross‐lagged framework. Two models were estimated, utilizing both T1 insomnia (model 1) and T2 insomnia (model 2) as intermediary variables. Although both models yielded statistically significant indirect effects (model 1 indirect *β* = 0.027, *p* = 0.007; model 2 indirect *β* = 0.008, *p* = 0.036), these results should be interpreted cautiously. In a two‐wave design, indirect effects may arise under multiple plausible processes, including reciprocal relations among constructs, changes occurring between assessment points, or the influence of unmeasured time‐varying factors. Consequently, the indirect effects reported here should be viewed as descriptive associations rather than evidence of mediation, temporal ordering, or intervening mechanisms.

## 3 Results

Anxiety, insomnia, and depressive symptoms were treated as continuous variables in all analyses. No clinical thresholds or diagnostic cutoffs were applied, as the purpose of the analyses was to examine prospective associations across the full range of symptom severity rather than to classify participants into diagnostic categories. Therefore, all models were estimated using continuous scores from self‐report measures of anxiety, insomnia and depression. Categorization related to thresholds for clinically elevated symptoms is provided for descriptive purposes only (to characterize the nature of the sample being studied).

### 3.1 Descriptives

At time 1, 15.1% of participants reported clinically elevated insomnia symptoms (ISI ≥ 15), though 48.8% of participants reported at least subthreshold insomnia symptoms (ISI ≥ 8). 47.0% of participants reported clinically elevated levels of anxiety symptoms (STAI ≥ 40) and 46.3% reported clinically elevated depression symptoms (CES‐D ≥ 16). At time 2, these rates were slightly lower for insomnia symptoms (13.3%, ISI ≥ 15; 42.3%, ISI  8) and anxiety symptoms (40.2%, STAI ≥ 40), but increases in depressive symptoms were observed (62.9%, CES‐D ≥ 16). Means, standard deviations, and bivariate correlations among the study variables are presented in Table 1. All variables were significantly correlated with all other variables within and across time points (*r* values ranged from 0.44 to 0.73).

**Table 1:** Means, standard deviations, and correlations with confidence intervals.

Variable	*M*	SD	1	2	3	4	5
1. T1 ISI	8.19	5.90					
2. T1 STAI	39.77	11.84	0.52**				
			[0.49, 0.55]				
3. T1 CESD	16.71	12.09	0.63**	0.79**			
			[0.60, 0.65]	[0.78, 0.81]			
4. T2 ISI	7.46	5.81	0.73**	0.44**	0.52**		
			[0.71, 0.75]	[0.41, 0.47]	[0.49, 0.55]		
5. T2 STAI	37.87	11.70	0.46**	0.68**	0.66**	0.55**	
			[0.43, 0.49]	[0.66, 0.70]	[0.63, 0.68]	[0.52, 0.57]	
6. T2 CESD	19.28	7.23	0.49**	0.52**	0.64**	0.60**	0.69**
			[0.46, 0.52]	[0.49, 0.54]	[0.62, 0.66]	[0.57, 0.62]	[0.67, 0.71]

*Note: M* and SD are used to represent mean and standard deviation, respectively. Values in square brackets indicate the 95% confidence interval for each correlation. The confidence interval is a plausible range of population correlations that could have caused the sample correlation (Cumming 2014).

** indicates *p* < 0.01.

### 3.2 Cross‐Lagged Model

To test the study hypotheses, an auto‐regressive cross‐lagged model was estimated with three predictors (T1 ISI, STAI, and CESD scores) and three outcome variables (T2 ISI, STAI, and CESD scores). All T2 variables were regressed onto T1 variables. Predictors were covaried. The model was fully saturated (0 degrees of freedom), meaning that all possible paths among the variables were estimated. As a result, overall model fit could not be evaluated, as saturated models do not permit estimation of goodness‐of‐fit indices. Accordingly, interpretation of the findings was based on the individual parameter estimates (i.e., path coefficients and their statistical significance) rather than on overall model fit or comparisons among alternative model specifications.

Controlling for T1 depression symptoms (*β* = 0.535, *p* < 0.001), T1 insomnia symptoms (H3; *β* = 0.142, *p* < 0.001), but not T1 anxiety symptoms (H1; *β* = 0.019, *p* = 0.445), significantly predicted T2 depression symptoms. Controlling for T1 insomnia symptoms (*β* = 0.661, *p* < 0.001), T1 depression symptoms (*β* = 0.077, *p* = 0.001), but not T1 anxiety symptoms (H2; *β* = 0.039, *p* = 0.075), significantly predicted T2 insomnia symptoms. Finally, controlling for T1 anxiety symptoms (*β* = 0.423, *p* < 0.001), both T1 insomnia symptoms (*β* = 0.060, *p* = 0.001) and T1 depression symptoms (*β* = 0.281, *p* < 0.001) significantly predicted T2 anxiety symptoms. Overall, insomnia symptoms prospectively predicted both subsequent depressive symptoms and anxiety symptoms, whereas anxiety symptoms did not significantly predict later insomnia or depressive symptoms after accounting for baseline symptom levels. Depression symptoms also prospectively predicted both later anxiety symptoms and insomnia symptoms, with the largest cross‐lagged effect observed for the association between depression and subsequent anxiety (*β* = 0.281). Stability coefficients between T1 and T2 were significant for anxiety symptoms (*β* = 0.423, *p* < 0.001), insomnia symptoms (*β* = 0.661, *p* < 0.001), and depression symptoms (*β* = 0.535, *p* < 0.001). In addition to the hypothesized cross‐lagged effects, other cross‐lagged effects are reported in Tables 2 and 3. Results of the hypothesized paths are described above and represented in Figures 1 and 2. In addition to these primary effects, the fully saturated model estimates all other possible cross‐lagged paths, which are reported in Table 3. Table 3 therefore provides a comprehensive view of exploratory associations among the study variables, beyond the specific hypotheses. These paths should be interpreted as descriptive and exploratory; they were not the focus of the study hypotheses and are presented to provide a complete representation of the model.

**Table 2:** Standardized regressions for study variables.

Variable	Std. all	*p‐*value
Insomnia T2		
Anxiety T1	0.039	0.075
Depression T1	0.077	0.001
Insomnia T1	0.661	< 0.001
Depression T2		
Anxiety T1	0.019	0.445
Depression T1	0.535	< 0.001
Insomnia T1	0.142	< 0.001
Anxiety T2		
Anxiety T1	0.423	< 0.001
Depression T1	0.281	< 0.001
Insomnia T1	0.060	0.001

**Table 3:** Standardized covariances for study variables.

Variable	Std. all	*p‐*value
Anxiety T1		
Insomnia T1	0.519	< 0.001
Depression T1	0.795	< 0.001
Depression T1		
Insomnia T1	0.628	< 0.001
Insomnia T2		
Depression T2	0.399	< 0.001
Anxiety T2	0.348	< 0.001
Depression T2		
Anxiety T2	0.488	< 0.001

### 3.3 Indirect Effect of Insomnia on the Anxiety‐Depression Link

As a secondary exploratory analysis, indirect effects linking T1 anxiety symptoms and T2 depressive symptoms through insomnia symptoms were estimated. Two models were examined. The first utilized T1 insomnia symptoms as the intermediary variable (Figure 1), whereas the second utilized T2 insomnia symptoms (Figure 2). In both models, the indirect effect linking anxiety symptoms at T1 with depression symptoms at T2 was statistically significant (model 1 indirect *β* = 0.027, *p* = 0.007; model 2 indirect *β* = 0.008, *p* = 0.036). Consistent with recommendations regarding two‐wave longitudinal designs, these indirect effects should not be interpreted as evidence of mediation, temporal sequencing, or underlying mechanisms. Rather, they represent descriptive associations derived from algebraic combinations of estimated regression paths within the cross‐lagged framework.

**Figure 1:**
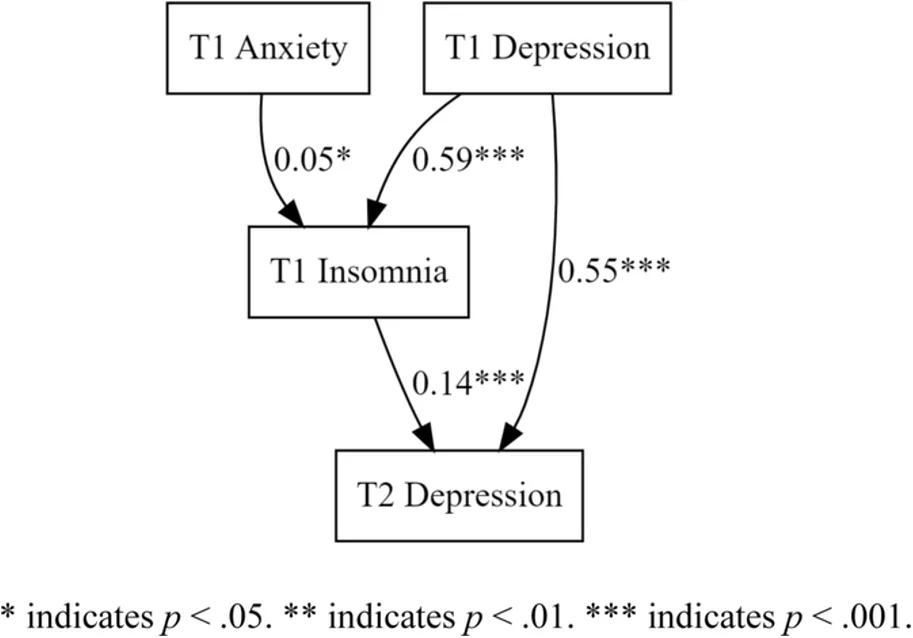
Mediation model with T1 insomnia as mediator. * indicates *p* < 0.05. ** indicates *p* < 0.01. *** indicates *p* < 0.001.

**Figure 2:**
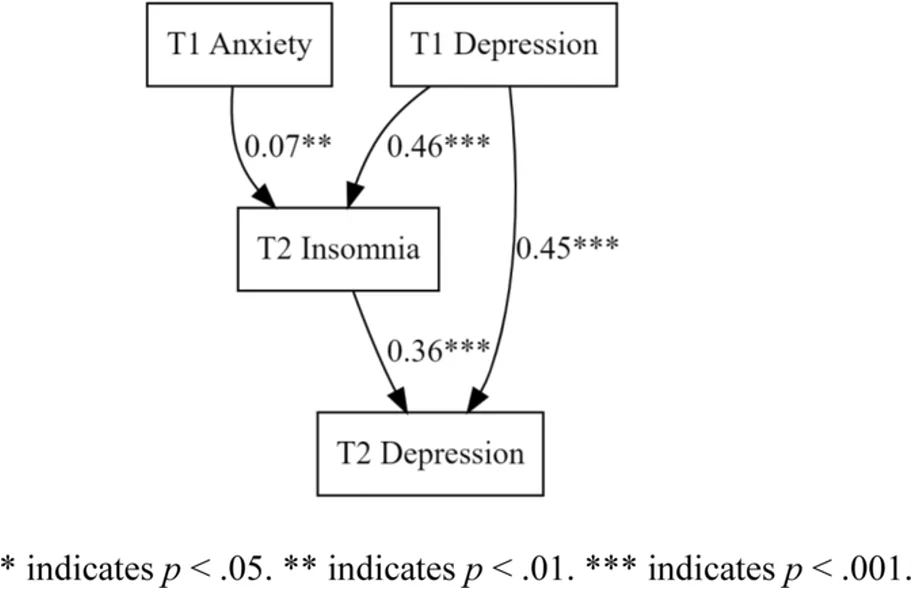
Mediation model with T2 insomnia as mediator. * indicates *p* < 0.05. ** indicates *p* < 0.01. *** indicates *p* < 0.001.

## 4 Discussion

The goal of this study was to examine prospective associations among insomnia, anxiety, and depression symptoms during the COVID‐19 pandemic. The primary finding was that insomnia symptoms prospectively predicted both depressive symptoms and anxiety symptoms over time. Depressive symptoms also predicted subsequent anxiety symptoms and, to a lesser extent, insomnia symptoms. In contrast, anxiety symptoms did not significantly predict later insomnia or depressive symptoms after accounting for baseline symptom levels. Together, these findings highlight prospective associations among insomnia, anxiety, and depressive symptoms and underscore the complex longitudinal relations among these symptom domains.

The spacing between assessments was determined primarily by the timing of the COVID‐19 pandemic rather than by an empirically based interval. Baseline data were collected during the initial onset of the pandemic, whereas follow‐up data were obtained approximately 1 year later, after the acute phase and early adjustment period had passed. This interval was selected to ensure that sufficient time had elapsed to observe meaningful changes in anxiety, insomnia, and depressive symptoms, rather than short‐term fluctuations associated with immediate stress responses. Notably, prior research suggests that elevations in anxiety and depressive symptoms during the COVID‐19 pandemic stabilized within several months for many individuals, supporting the use of a longer follow‐up period to capture more sustained symptom trajectories

To better situate our results within the broader literature, it is important to emphasize that the central contributions of the present study come from the specific temporal patterns that emerged. Our findings indicate that insomnia symptoms prospectively predicted both anxiety and depressive symptoms. This pattern aligns with, and extends, previous research demonstrating the effects of sleep disturbance on mood and anxiety symptoms, and the present study provides direct longitudinal evidence for this pattern in a large, population‐based adult sample assessed during a period of elevated stress. We also found that depressive symptoms predicted later anxiety symptoms, which is a notable result that contributes to ongoing debates about bidirectionality among internalizing symptoms. By highlighting the temporal relations supported by our own analyses, the present study helps clarify which pathways appear most robust in this dataset and how they converge with, or diverge from, prior theoretical frameworks.

Notably, depressive symptoms at T1 significantly predicted increases in anxiety symptoms at T2, and this path represented one of the larger cross‐lagged effects in the model (*β* = 0.281). This finding is consistent with conceptual models suggesting that depressive symptoms may heighten vulnerability to subsequent anxiety through mechanisms such as increased rumination, threat sensitivity, and impaired emotion regulation. These results underscore the dynamic relationship among symptoms and highlight the value of assessing and intervening on depressive symptoms early to potentially mitigate downstream anxiety symptoms.

As a secondary exploratory analysis, indirect effects linking anxiety symptoms and later depressive symptoms through insomnia symptoms were estimated. Significant indirect effects were observed in both models. However, because the present study included only two assessment points, these effects should not be interpreted as evidence of mediation, temporal sequencing, or causal mechanisms. Similar indirect effects may arise under multiple plausible processes, including reciprocal symptom dynamics, changes occurring between assessment occasions, or the influence of unmeasured time‐varying factors. Consequently, these findings are best viewed as descriptive associations that may inform future multi‐wave investigations rather than evidence supporting a specific developmental pathway.

Beyond the overall pattern observed in the present study, it is important to acknowledge that the temporal ordering among insomnia, anxiety, and depression may vary across individuals and contexts. Although our findings indicate that insomnia prospectively predicted subsequent anxiety and depression, prior work suggests substantial heterogeneity in how these symptoms develop over time. Individual differences in stress responsivity, emotion regulation, developmental stage, or genetic vulnerability, as well as contextual factors such as major life stressors or health changes, may alter the sequence in which symptoms emerge. For some individuals, anxiety may initiate sleep disruption, whereas in others, depressive symptoms may precede both disturbed sleep and anxiety. These possibilities highlight that symptom clusters can influence one another in multiple directions. Recognizing this heterogeneity underscores the complexity of these dynamic processes and highlights the need for future research using more frequent longitudinal assessments to identify person‐specific pathways.

### 4.1 Limitations

The COVID‐19 pandemic was a possible confound in this study. Gathering data during this historical event presents the risk that the characteristic links that were found between variables are not going to be present moving forward. This potential discrepancy could be explained by the impact of unique environmental factors related to the COVID‐19 pandemic during the time of data collection. Although the general impact of the pandemic on sleep was an increase in insomnia (Morin et al. 2021), the actual quality and duration of sleep parameters are more variable. For example, one study found that the effect of the pandemic on sleep differs across individuals and depends on their pre‐pandemic sleep quality (Kocevska et al. 2020). Specifically, some individuals with pre‐pandemic insomnia actually experienced an improvement in the quality of their sleep, while pre‐pandemic good sleepers most often experienced worse sleep during times of pandemic lockdown. Another consideration is that sleep duration increased for many individuals during the pandemic (Benham 2021; Trakada et al. 2020). This change could be partially attributed to the common transition to working from home, which meant later wake times as a result of the time saved by no longer commuting to work. One study found that bedtime and waketime were significantly later during the pandemic compared to pre‐pandemic times (Benham 2021). It is known that short sleep duration is associated with depression (Furihata et al. 2015), so it stands to reason that increasing sleep duration to a healthier level might serve as a protective factor against new onset depression. Considering that one may meet criteria for insomnia regardless of their sleep duration, it is plausible that sleeping longer could have masked the impact of insomnia on the development of depression symptoms. Furthermore, there were also findings of greater sleep medication use during the pandemic (Benham 2021) which could have prevented the progression of insomnia symptoms into the development of depression.

Another limitation of this study was that data were collected at only two time points, which required more complex analyses to determine the temporal order of the three variables. These analyses involved cross‐sectional (as well as longitudinal) paths within each model, and required multiple models in order to include temporal ordering at every path. Ideally, when testing the temporal order of three variables, there would be at least three time points to test mediation. That said, these data provide initial longitudinal evidence regarding prospective associations among anxiety, insomnia, and depression. To get more detailed information about the progression of these symptoms over time (e.g., the relative impact of acute vs. chronic or new onset vs. persistent symptoms), future efforts should use a high‐density sampling approach (e.g., weekly or monthly measurements for 1+ years).

It is important to note that the use of only two time points inherently assumes that the temporal lag between constructs is adequately captured by the interval between assessments. This assumption is difficult to verify and may not hold uniformly across individuals or contexts. Symptoms of anxiety, insomnia, and depression may unfold over shorter or longer time spans than the 9–12 month lag used in this study, and the optimal temporal spacing for detecting directional effects likely varies across people, developmental stages, and environmental conditions. As a result, our findings reflect average longitudinal associations across the sample rather than precise person‐level temporal dynamics. Although the cross‐lagged approach provides information about temporal precedence at the group level, future research with higher‐frequency or multi‐wave designs is needed to determine how quickly these symptom processes influence one another and to identify potential individual differences in lagged effects. Furthermore, shorter intervals would allow for greater temporal resolution, making it possible to observe transient symptom fluctuations, detect lagged effects, and distinguish between acute versus chronic changes in symptoms. In contrast, the current study used a longer interval of approximately 9–12 months between assessments, which provides valuable information about longer‐term trends but may miss shorter‐term dynamics that unfold over days or weeks. As such, our findings primarily reflect cumulative effects rather than fine‐grained temporal processes.

Another important consideration is the omission of stress from the current models. Stress is a well‐established contributor to both insomnia and mood symptoms, and prior work has demonstrated that elevated stress can precipitate sleep disturbance as well as increases in anxiety and depression. Because our analyses did not include a measure of perceived stress, it is possible that stress may account for some of the observed associations among insomnia, anxiety, and depression. For example, stress could operate as a shared factor influencing all three constructs or as a time‐varying confound that alters the apparent temporal ordering of symptoms. It is also possible that the differential effects that the pandemic had on people (and their stress levels) may also play a particularly relevant role in the sequencing of symptom presentation. Although the present data did not allow us to explicitly test these possibilities, acknowledging this limitation is important for interpreting the directionality of effects. Future studies incorporating repeated assessments of stress would help clarify the degree to which stress contributes to or modifies the dynamic interplay among these symptoms.

Lastly, although this study was conducted during the COVID‐19 pandemic, we assessed anxiety using the State‐Trait Anxiety Inventory (STAI), a general measure of anxiety symptoms, rather than a COVID‐19–specific scale (e.g., Coronavirus Anxiety Scale [Lee 2020], Stress and Anxiety to Viral Epidemics‐9 [Chung et al. 2021], Fear of COVID‐19 Scale [Blazquez et al. 2022]). In addition, we did not include measures of pandemic‐specific stressors, such as exposure risk, quarantine or isolation status, or employment and financial impacts. As a result, we cannot determine the extent to which the anxiety, insomnia, or depression symptoms observed were specifically related to the pandemic versus broader contextual or individual factors. Future research incorporating virus‐specific anxiety measures and COVID‐related stressors would help clarify the pandemic‐specific aspects of these associations and determine how the relationships among anxiety, insomnia, and depression may differ from those observed in non‐pandemic contexts.

In summary, insomnia symptoms prospectively predicted both anxiety and depressive symptoms over time, whereas anxiety symptoms did not predict subsequent insomnia or depression after accounting for baseline symptom levels. Although the observed prospective associations were statistically significant, their modest effect sizes suggest that the findings are most informative for understanding longitudinal symptom relationships rather than indicating large clinical effects. These findings highlight the potential importance of sleep disturbance within the broader network of internalizing symptoms and suggest that sleep may represent one potential target for prevention and early intervention efforts. Additional multi‐wave longitudinal studies are needed to clarify the mechanisms and temporal dynamics linking insomnia, anxiety, and depression.

## Ethics Statement

This study was approved by the University of Arkansas Institutional Review Board (IRB), Approval No. 200325853.

## Conflicts of Interest

The authors declare no conflicts of interest.

## Data Availability

The data that support the findings of this study are openly available in Data Repository at https://sleeplab.nd.edu/resources/research-resources/open-science/.
